# Vegetation Restoration in Karst Southwest China: Effects of Plant Community Diversity and Soil Physicochemical Properties on Soil Cadmium

**DOI:** 10.3390/toxics14010102

**Published:** 2026-01-21

**Authors:** Yun Xing, Lin Zhang, Zhuoyi Mei, Xiuwen Wang, Chao Li, Zuran Li, Yuan Li

**Affiliations:** 1 College of Animal Science and Technology, Yunnan Agricultural University, Kunming 650201, China; xingyun654813787@sohu.com (Y.X.); zhanglin3039@163.com (L.Z.); 2 College of Resources and Environment, Yunnan Agricultural University, Kunming 650201, China; 14780083690@163.com (Z.M.); Wangxw424@163.com (X.W.); 18314551926@163.com (C.L.); 3 Yunnan Center for Disease Control and Prevention, Yunnan Institute of Preventive Medicine, Kunming 650022, China; 4 College of Horticulture and Landscape, Yunnan Agricultural University, Kunming 650201, China

**Keywords:** cadmium, karst, Yilong lake, vegetation restoration

## Abstract

In southwest China, vegetation restoration is widely used in karst rocky desertification control projects. However, mechanistic evidence explaining how plant community composition and species diversity regulate cadmium (Cd) bioavailability remains limited. Here, the plant community’s species diversity, soil properties, Cd, and available Cd contents were evaluated. Four plant community types, NR (natural recovery), PMC (*Pistacia weinmannifolia* + *Medicago sativa* + *Chrysopogon zizanioides*), and PME (*Pistacia weinmannifolia* + *Medicago sativa* + *Eragrostis curvula*), were selected as the research objects. The species composition was recorded, and dominant plant species and soil samples were collected to analyze Cd accumulation characteristics. Relative to NR, composite restorations increased plant diversity and soil nutrient availability and reduced soil compaction, with PMC showing the strongest remediation, decreasing total Cd by 49.4% and available Cd by 59.5%. Model-averaged regression and hierarchical partitioning analyses further identified nitrogen availability and community structure as the dominant drivers. Specifically, available nitrogen (AN), vegetation coverage, Margalef species richness (DMG), ammonium nitrogen (NH_4_^+^–N), and total N (TN) were the main factors of soil total Cd, and BD, TN, nitrate nitrogen (NO_3_^−^–N), mean crown diameter (MCD), and Shannon–Wiener index (*H*′) were the main factors of soil available Cd. The results indicate that PMC provides a plant community structure configuration decisions of a scalable, site-adaptable strategy for durable Cd stabilization and soil conservation in thin, carbonate-rich karst soils.

## 1. Introduction

Cadmium (Cd) stands out as the most hazardous heavy metal, displaying a non-degradable nature and an extended half-life in soil [1]. Excessive accumulation of heavy metals in soils can deteriorate soil fertility and reduce its productive capacity [2]. It can harm soil microbes and plant growth, and may pose a threat to human health through the food chain [3]. Cd poses long-term risks to ecosystem functioning and human health due to its bioaccumulative nature [4]. These risks can be categorized into two types: anthropogenic contamination and naturally high background regions [5].

Karst regions characterized by high geochemical backgrounds exemplify the latter, where rock types, diagenetic environments, and tectonic activity have resulted in markedly enriched levels of heavy metal content in soils and rocks, particularly in carbonate rock formations [6]. Compared with non-karst areas, karst ecosystems are highly fragile, owing to their weak rock–soil system and coupled surface–subsurface hydrological framework [7]. Recent evidence further indicates that karst systems are exceptionally sensitive to climate extremes, including intensified diurnal temperature ranges and altered wind regimes, which can amplify soil moisture fluctuations and erosion processes [8]. Under such climate stress, vegetation cover becomes a critical regulator of surface–subsurface hydrological connectivity, thereby influencing Cd mobilization and lateral transport in karst soils [9]. Research in high-geochemical-background regions highlights that the high Cd content is mainly derived from the parent rock [10]. Vegetation restoration has been widely applied in southwest China to mitigate rocky desertification. This approach improves soil aeration and porosity, enhances soil nutrient levels, and reduces heavy metal concentrations, thereby promoting the recovery of ecosystem structure and function. [3,11]. Multiple studies have reported that vegetation recovery can substantially reduce both total and extractable Cd in karst soils [3,12,13]. Other studies have reported different degrees of soil heavy metal pollution across different land utilization and vegetation categories within karst areas [14,15]. Vegetation restoration not only conserves soil and water resources and reduces soil erosion, but also improves soil quality via soil–plant interaction, which is an essential measure for rocky desertification management and ecological reconstruction [16]. Plant community diversity can influence soil Cd content and bioavailability through vegetation-driven changes in the cover, biomass, and litter inputs that affect nutrient cycling [17,18]. It can also influence soil bulk density [19], porosity, organic matter, and nitrogen status, thereby shifting Cd between solid-bound and extractable forms [20,21]. A previous study showed that shrub ecosystems in karst areas exhibit a greater capacity for improving soil quality, thereby promoting the restoration of ecosystem structure and function [22]. Current studies on soil heavy metal contamination in karst regions of southwest China mainly focus on the effects of heavy metal pollution on soil properties [23] and the distribution and remediation [24] of heavy metals under different land-use types [25]. However, most research focuses on single-species responses, and mechanistic evidence on how plant community composition and species diversity influence Cd mobility and extractability in karst soils is limited [26]. Therefore, it is essential to investigate how plant communities regulate soil total and bioavailable Cd by modulating physical structure, soil chemical properties, plant uptake, and functional–complementarity pathways in karst areas.

**Figure 1:**
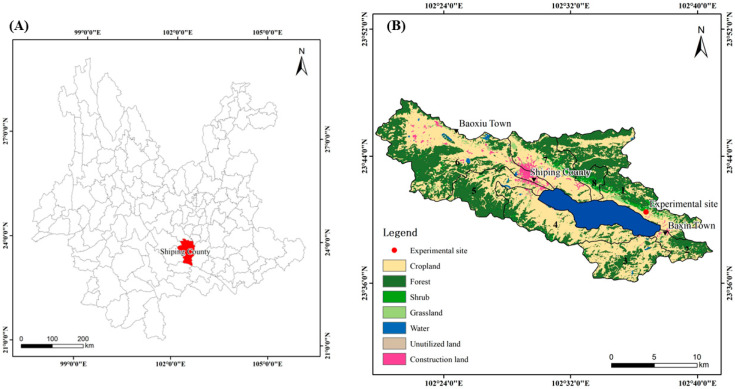
Location and overview of the experimental site. (**A**) Geographic location of Shiping County in Yunnan Province. (**B**) Land-use distribution map of the Yilong Lake Basin. The red dot indicates the location of the experimental site. The numbers (1–8) indicate different sub-catchments within the Yilong Lake Basin, including the northern lakeshore dispersal area (1), Yucun River sub-basin (2), Longgang River sub-basin (3), southern lakeshore dispersal area (4), Chengnan River sub-basin (5), Cheng River sub-basin (6), Chengbei River sub-basin (7), and Dashui River sub-basin (8).

The karst area of southwest China is characterized by a subtropical monsoon climate with ample rainfall and thermal resources, though these are irregularly distributed spatially and temporally [27]. The area is also characterized by high rock exposure rates, thin and discontinuous soil layers, and sticky and heavy soil textures of low fertility [28]. The Yilong Lake Basin, situated in Shiping County, south-central Yunnan (23°36′–23°48′ N, 102°20′–102°41′ E; Figure 1), spans 360.4 km^2^, with an average elevation of 1414 m above sea level [29]. The basin experiences a subtropical monsoon climate, characterized by a distinct dry season (November–June) and wet season (July–October), with a mean annual precipitation of 928.3 mm, evapotranspiration of 1908.6 mm, and an average temperature of 18 °C. The Yilong Lake watershed primarily comprises Yilong, Baoxiu, and Bazin towns of Shiping County, extending in an east–west orientation [30]. The principal soil types are Dystric Cambi soils, Cumulic Anthro soils, Haplic Acri soils, and Humic Acri soils [30]. Accordingly, the northern lakeshore area of Baxin Town, a representative karst region surrounding Yilong Lake, was selected as the study site to (1) analyze the effects of different restoration measures on community characteristics, soil physicochemical properties, and the concentrations of total and available cadmium in soils, and (2) quantify relationships among plant community structures, soil physical properties, soil chemical factors, and Cd bioavailability. This study provides practical recommendations for ecological restoration in karst areas. It offers perspectives to improve restoration strategies and enhance the efficiency of eco-engineering efforts, enhancing ecological engineering efficiency while promoting the control, stabilization, and sustainable use of Cd-contaminated soils in karst regions, with potential applicability to similar environments worldwide.

## 2. Materials and Methods

### 2.1. Study Area

The experimental site of this study is located on the northern shore of Yilong Lake, Baxin Town, Dalongjing Mountain, Shiping County, Yunnan Province, China (23°40′12″ N, 102°36′36″ E) (Figure 1). The area represents a karst mountainous landscape characterized by sparse vegetation and severe soil erosion, with an elevation of 1452 m. The region experiences a subtropical monsoon climate, with a mean annual temperature of 18.3 °C, average annual sunshine duration of 2145.2 h, and annual precipitation ranging between 786 mm and 1116 mm. Rainfall varies greatly among years, with abundant sunlight throughout the four seasons, concentrated precipitation in summer and autumn, and relatively little rainfall in winter. The mountain soil is primarily sandy. The vegetation in the mining area is sparse, mainly including shrubs and herbs. The dominant plants include *Imperata cylindrica* (L.) Raeusch., *Miscanthus sinensis* Anderss., *Bidens pilosa* L., *Heteropogon contortus* (L.) Beauv. ex Roem. & Schult., *Pistacia weinmanniifolia* J. Poiss., *Leucaena leucocephala* (Lam.) de Wit, *Photinia serratifolia* (Desf.) Kalkman, and *Salix myrtillacea* Andersson.

**Figure 2:**
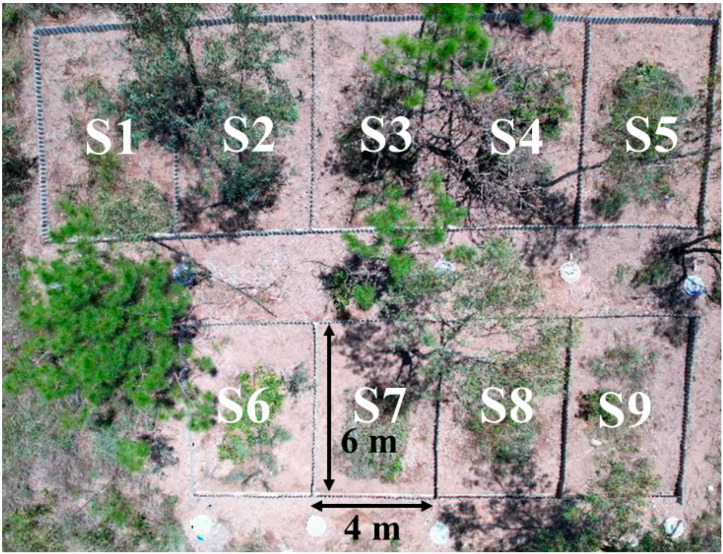
Aerial photograph of the experimental plots used for vegetation restoration.

According to the soil conditions of the northern bank of Lake Yilong and the physiological characteristics of the selected plants, *Pistacia weinmannifolia* J. Poiss., *Medicago sativa* cv. WL525HQ, *Chrysopogon zizanioides* (L.) Roberty, and *Eragrostis curvula* (Schrad.) Nees were chosen to establish a two-layer “shrub + herb” restoration model. Seeds of *Medicago sativa* and *Eragrostis curvula* were purchased from Beijing Zhengdao Seed Co., Ltd. (Beijing, China), while seedlings of *Pistacia weinmannifolia* and *Chrysopogon zizanioides* were obtained from the Kunming Zhengxin Seedling Base (Kunming, China). The experimental site consisted of nine plots (each 4 m × 6 m) with slopes of 22–26°. Plots were separated by asbestos boards buried underground to prevent hydrological interference between adjacent plots (Figure 2). To minimize potential edge effects and hydrological interference, asbestos boards were installed between adjacent plots and buried belowground.

Three planting models were established: PMC (*Pistacia weinmannifolia* + *Medicago sativa* + *Chrysopogon zizanioides*), PME (*Pistacia weinmannifolia* + *Medicago sativa* + *Eragrostis curvula*), and a control treatment (NR) representing the original natural shrub–grass community under natural growth without intervention. *Pistacia weinmannifolia* individuals with similar growth vigor were selected and planted at a spacing of 1.5 × 1 m. Planting and seeding were completed in June 2022. *Chrysopogon zizanioides* was interplanted between shrub rows in nine planting holes per plot, with five seedlings per hole. *Medicago sativa* and *Eragrostis curvula* were sown as a mixture (1:1 ratio) with a seeding rate of 2 kg per 666.7 m^2^ and a row spacing of 30 cm, ensuring good seed–soil contact. Soils were tilled and leveled before planting to ensure uniform conditions. Each model was replicated three times. Plot arrangements are shown in Table 1.

*P. weinmanniifolia* J. Poiss, a shrub species belonging to the family Anacardiaceae, is native to Yunnan Province, China. It typically grows in limestone mountain forests or shrublands and is a heliophilous species [31]. *M. sativa* L. is a perennial leguminous herb with strong ecological adaptability and well-developed root system possesses nitrogen-fixing capability, thereby enhancing soil fertility [32,33]. *C. zizanioides* (L.) Roberty, a gramineous xerophytic herb, is characterized by rapid growth, an extensive root system, and strong slope-stabilization capacity. This species exhibits broad environmental adaptability, high stress tolerance, and the ability to thrive in nutrient-poor soils [34]. *E. curvula* (Schrad.) Nees is a perennial grass species in the Poaceae family, known for its high drought tolerance and resistance to soil infertility. Owing to its extensive root system and strong regenerative capacity, it commonly colonizes disturbed or degraded sites and plays an important role in vegetation restoration [35].

### 2.2. Analysis of Community Traits

Vegetation investigations were conducted in October 2024, recording shrubs and herbs in each plot. Three 1 × 1 m quadrats were randomly selected per plot [36]. For shrub and grass layers, the number of individuals, average height, and canopy width were measured in each quadrat. Height and canopy width were measured with tape lines. Vegetation cover is the percentage of the vertical projection of the plant parts; slope was measured with a handheld GPS device placed on the ground. Species identification was based on regional floras and the Flora República Popularis Senica website [37]. Taxonomic identification was conducted using field classification, literature manuals and monographs, and professional expert identification by the Kunming Institute of Botany. Plant coverage of each plot was visually estimated by three investigators. Basic plot information is shown in Table 2.

### 2.3. Sample Collection and Preparation

Soils at 0–20 cm were collected by the five-point method in each plot and mixed into one composite. In each plot, two dominant species were sampled, with two mature plants per species representing spontaneous growth. All plant and soil samples were sealed in clean PE bags and delivered to the laboratory [38]. After removing litter, a cutting ring 100 cm^3^ collected soils at 0–20 cm horizontally. Soils were naturally dried, ground, and crushed, passed through 20- and 100-mesh sieves, and sealed at 0–4 °C. Plant samples were divided into shoots and roots. The soil attached to the plant samples was rinsed off with tap water, and subsequently the plants were washed with deionized water 2–3 times. Shoots and roots were deactivated at 70 °C for 30 min, dried at 55 °C to constant weight, pulverized, passed through 100-mesh sieves, and stored sealed for subsequent analysis [39].

### 2.4. Soil and Plant Chemical and Metal Analysis

BD, non-capillary porosity (NCP), capillary porosity (CP), and total porosity (TPO) were determined by the cutting ring method [40]. Soil organic matter (SOM) was determined by the sulfuric acid–potassium dichromate method (NY/T 1121.6-2006). Total nitrogen (TN) was measured by the Kjeldahl method. Total phosphorus (TP) was determined by the molybdenum–antimony colorimetric method. Available nitrogen was determined by the alkaline hydrolysis diffusion method, and available phosphorus was measured by atomic absorption spectrophotometry method (PerkinElmer Inc., Waltham, MA, USA) [40]. Available Cd was measured by CaCl_2_ extraction and atomic absorption spectrometry [41]. Soil samples were digested in an acid mixture of HNO_3_–HClO_4_–HF (5:4:1) with a graphite digestion oven (CEM Corporation, Matthews, NC, USA) [42]. Plant Cd was determined through the HNO_3_–HClO_4_ mixed-acid method. Cd in digests was quantified by graphite furnace atomic absorption; method setup followed HJ 832-2017 and GB 5009.15-2014. The LODs for soil and plant Cd were 0.01 mg·kg^−1^ and 0.02 mg·kg^−1^, respectively. All samples were analyzed in triplicate, and the relative standard deviation (RSD) of replicate measurements was less than 5%. No significant contamination was detected in blank samples. Two calibration standards were tested for every 10 samples, and the calibration curves showed coefficients of determination (R^2^) greater than 0.990.

### 2.5. Calculation Methods for Species Diversity and Accumulation Characteristics

Species diversity parameters were calculated as follows [43]: Ecological dominance (*Do*), Margalef species richness (*DMG*), Shannon–Wiener index (*H*′), Simpson’s diversity index (*Dsi*), and Pielou’s evenness index (*Epi*).

(1) $$Ecological\;Dominance\;\left(Do\right)=\sum_{i=1}^{S}\frac{n_{i}\left(n_{i}-1\right)}{N\left(N-1\right)}$$

(2) $$Margalef\;Species\;Richness\;\left(DMG\right)=\frac{S-1}{\ln N}$$

(3) $$Shannon\;Wiener\;Index\;\left(H^{\prime }\right)\;=\;-\sum_{i=1}^{S}P_{i}\;\ln P_{i}$$

(4) $$Simpson\text{’}s\;Diversity\;Index\;\left(Dsi\right)=1-\sum p_{i}^{2}$$

(5) $$Pielou\;Evenness\;Index\;\left(Epi\right)=\frac{H^{\prime }}{\ln S}$$

In these equations, *S* is the total number of species, *n_i_* is the number of individuals of the *i*-th species, *N* represents the total number of individuals, and *P_i_* = *n_i_*/*N* denotes the relative abundance of the *i*-th species. The natural logarithm (ln) is used in all logarithmic calculations.

The importance value (IV) of each plant species is calculated as follows [44]:

(6) $$\mathrm{Importance}\;\mathrm{value}\;(\mathrm{IV})=\frac{\mathrm{RD}+\mathrm{RC}}{2}$$

In this equation, *RD* is the relative density of species and *RC* is the relative coverage of the species. Species with the highest IVs within each treatment were identified as the dominant species and selected for subsequent biomass and cadmium accumulation analyses. Detailed IV values of all recorded species under different treatments are provided in Appendix A (Table A1).

The bioaccumulation factor (BCF) is determined by the ratio of heavy metal concentration in the shoot parts to that in the soil [45]. The translocation coefficient (TC) is calculated as the heavy metal concentration in shoots divided the concentration in roots [46].

### 2.6. Statistics Analysis

Differences in species richness, evenness index, soil physicochemical properties, total and available cadmium contents, and plant cadmium concentrations among different treatments were examined using one-way ANOVA. Pearson correlation coefficients (significance *p* < 0.05, very significant *p* < 0.01) were applied to examine the relationships between species diversity and soil properties, verified using the R packages spaa and corrgram (version 4.4.3). Variables that showed significant correlations with total and available cadmium in the Pearson correlation analysis were selected as predictor variables in the models. Multiple regression models were applied to evaluate the effects of species diversity and soil properties on total and available soil cadmium contents, respectively. All predictors and response variables were standardized using Z-scores to interpret parameters on a common scale. Using the “MuMIn” package [47], all possible combinations of the initial predictors were used to generate a set of candidate models. These models were ranked according to the Akaike information criterion (AIC) based on maximum-likelihood estimation in R. Models with ΔAIC < 2 were selected, and model averaging was applied to estimate parameters and associated *p*-values using the MuMIn function model.avg. The relative contribution of each independent variable was then calculated based on its parameter estimate relative to the total model effect. Given the limited number of replicates (*n* = 9), the regression and model averaging analyses were applied in an exploratory manner to evaluate the relative importance of candidate predictors, rather than to build predictive models.

## 3. Results

### 3.1. Plant Community Characteristics

**Figure 3:**
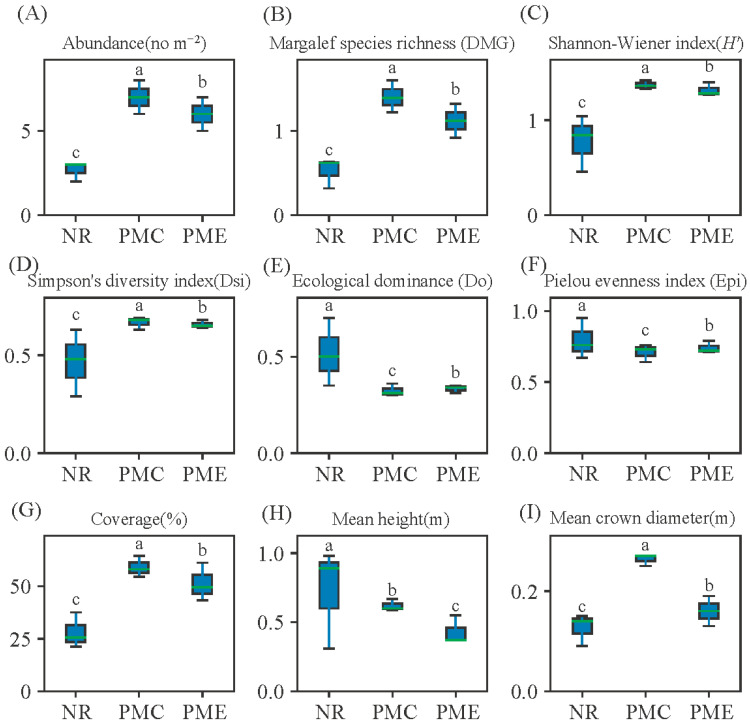
Characteristics of species diversity and plots profile of plant communities, Abundance of communities (**A**), Margalef species richness (**B**), Shannon–Wiener index (**C**), Simpson’s diversity index (**D**), Ecological dominance (**E**), Pielou evenness index (**F**), Vegetation coverage (**G**), Mean plant height (**H**), and Mean crown diameter (**I**). Data differences were examined using Duncan’s multiple range test, and bars with the same letter are not significantly different (*p* < 0.05, *n* = 9).

A total of 17 plant species belonging to 13 families and 15 genera are recorded across the experimental plots (Table 2). The dominant herbaceous species included *Imperata cylindrica* (L.) Raeusch., *Miscanthus sinensis* Anderss., *Bidens pilosa* L., *Medicago sativa* cv. WL525HQ, *Chrysopogon zizanioides* (L.) Roberty, and *Eragrostis curvula* (Schrad.). Plant abundance ranged from 23 to 93 individuals·m^−2^, showing higher values in the PMC and PME plots than in NR. Diversity parameters varied significantly among the three community types (Figure 3). Species abundance, richness (DMG), and Shannon–Wiener index (*H′*) were the highest in PMC, intermediate in PME, and the lowest in NR (*p* < 0.05). Conversely, ecological dominance (Do) was the greatest in NR, indicating that the community was mainly composed of a few dominant species. Simpson’s diversity index (Dsi), Pielou evenness index (Epi), coverage, mean height, and mean crown diameter followed the same pattern as richness and diversity, all showing significantly higher values in PMC than in NR (*p* < 0.05). Overall, the results suggest that vegetation restoration significantly increased plant abundance and diversity while reducing dominance. The community structure in PMC and PME plots shifted toward a more diverse and evenly distributed pattern compared with NR plots.

### 3.2. Soil Properties and Cd Concentrations

The physicochemical properties of soils in the three treatments (NR, PMC, and PME) are summarized in Table 3. BD differed significantly among treatments (*p* < 0.05), being the highest in NR (1.64 ± 0.12 g·cm^−3^) and the lowest in PMC (1.27 ± 0.06 g·cm^−3^). Available nitrogen (AN) and nitrate nitrogen (NO_3_^−^–N) followed the same pattern, while available phosphorus (AP) remained relatively stable across plots. Total and available Cd concentrations varied significantly among plots (*p* < 0.05), both being the lowest in PMC and the highest in NR. Specifically, total Cd decreased with a reduction of 49.4%, while PME showed a 35.3% decrease relative to NR. Similarly, available Cd declined from 0.042 ± 0.007 mg·kg^−1^ in NR to 0.017 ± 0.003 mg·kg^−1^ in PMC, representing a reduction of 59.5%, while a reduction of 35.7% was observed in PME compared with NR. These results indicate that vegetation restoration significantly reduced both total and bioavailable Cd in the soil, suggesting improved soil environmental quality under PMC conditions. Overall, PMC exhibited lower BD and Cd contents but higher nutrient availability, suggesting a potential improvement in soil structure and environmental quality.

### 3.3. Biomass Cd Content and Accumulation of Dominant Plants

**Figure 4:**
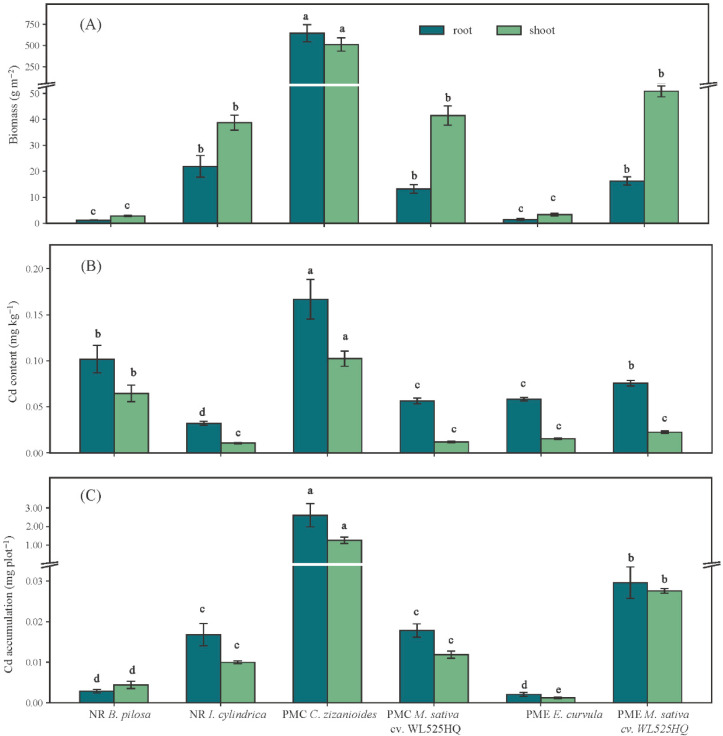
Biomass (**A**), Cd content (**B**), and Cd accumulation (**C**) in roots and shoots of dominant species of different treatments. The values are means ± SD of three replicates for each treatment. Different letters indicate significant differences among species at same plant parts according to Duncan at *p* < 0.05.

The highest biomass was observed in PMC *C. zizanioides* for both roots and shoots, whereas *B. pilosa* of NR and *E. curvula* of PME exhibited the lowest values. There was no significant difference in root and shoot biomass of *M. sativa* under PMC and PME treatments. The roots of PMC *C. zizanioides* contained the highest Cd concentration (0.167 ± 0.02 mg kg^−1^), while NR *I. cylindrica* had the lowest (0.032 ± 0.002 mg kg^−1^) (Figure 4B). Cd concentrations in roots were higher than those in shoots across all species. Cd accumulation (Figure 4C) exhibited a similar trend to content, with the greatest values of *C. zizanioides* in PMC of both root and shoot (*p* < 0.05). Cd accumulation (Figure 4C) exhibited a similar pattern to Cd content, with the greatest values observed in PMC *C. zizanioides* for both roots and shoots (*p* < 0.05) (2.603 ± 0.627 mg plot^−1^ and 1.255 ± 0.177 mg plot^−1^, respectively).

**Figure 5:**
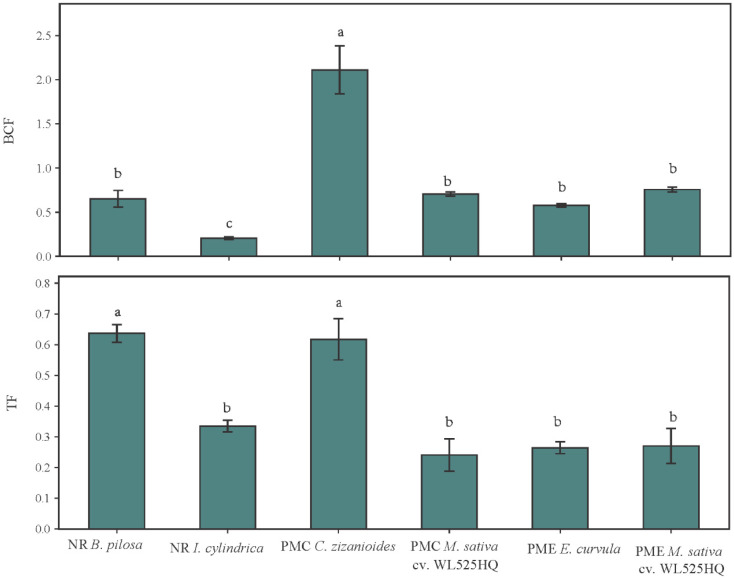
Bioconcentration factors (BCF) and translocation factors (TF) of Cd for dominant species of different treatments. Values are means ± SD of three replicates for each treatment. Different letters indicate significant differences among species at same plant parts according to Duncan at *p* < 0.05.

The bioconcentration factor (BCF) and translocation factor (TF) values of Cd for dominant species under different treatments are presented in Figure 5. The BCF of Cd ranged from 0.21 to 2.10 among all species, indicating large differences in Cd uptake ability. BCF and TF of Cd showed significant differences among the dominant species (*p* < 0.05). *C*. *zizanioides* in the PMC treatment exhibited the highest BCF (2.10 ± 0.23), suggesting a strong Cd accumulation potential in roots, while the lowest in NR *I. cylindrica*. There were no significant differences between *M. sativa* cv. WL525HQ of PMC, *E. curvula*, and *M. sativa* cv. WL525HQ of PME. The TF of Cd varied between 0.26 and 0.65 across the tested species. *B. pilosa* and *C. zizanioides* had the highest TF values with 0.65 ± 0.04 and 0.62 ± 0.05, respectively, both significantly greater than in others (*p* < 0.05), indicating efficient translocation of Cd from roots to shoots. In contrast, *I. cylindrica* (0.33 ± 0.02), *E. curvula* (0.29 ± 0.03), and *M. sativa* cv. WL525HQ (0.31 ± 0.04) had lower TF values, suggesting that most Cd was retained in the roots.

### 3.4. Correlations Between Environmental Variables and Cd Contents

**Figure 6:**
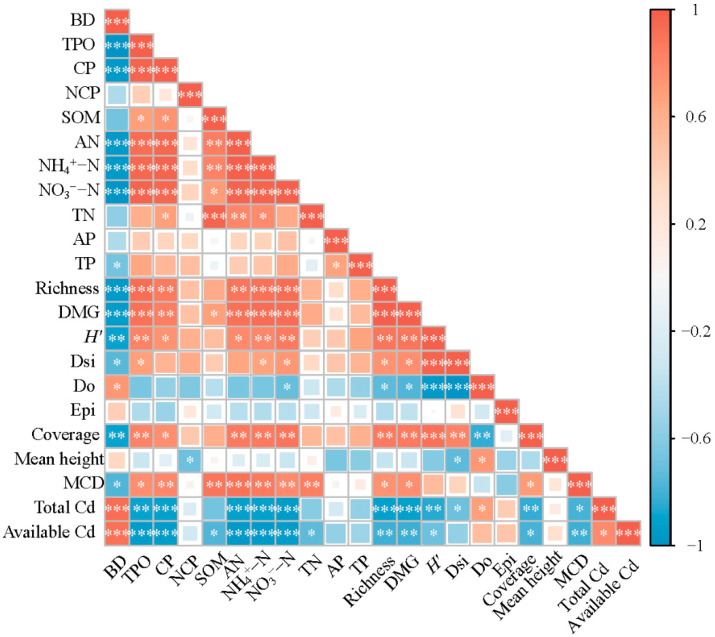
Pearson correlation coefficients among soil physicochemical properties (BD, TPO, CP, NCP, SOM, AN, NH_4_^+^–N, NO_3_^−^–N, TN, AP, TP), vegetation diversity parameters (Richness, DMG, *H*′, Dsi, Do, Epi, Coverage, Mean height, MCD), and cadmium concentrations (Total Cd, Available Cd). The color scale represents the correlation coefficients (red = positive correlation, blue = negative correlation). The size of the squares indicates the magnitude of the correlation coefficient. Only the lower triangular matrix is shown. Asterisks indicate significance levels: * (0.01 < *p* < 0.05), ** (0.001 < *p* ≤ 0.01), *** (*p* ≤ 0.001).

Species richness, DMG, *H*′, and coverage were positively correlated with AN, NH_4_^+^–N, NO_3_^−^–N (*p* < 0.05), indicating that diverse plant communities improved soil nutrient status (Figure 6). By contrast, these diversity variables were negatively correlated with BD (*p* < 0.05), suggesting that greater vegetation diversity enhanced soil porosity. Moreover, richness, DMG, coverage, mean crown diameter, and *H*′ showed negative correlations with Total Cd and Available Cd (*p* < 0.05), implying that higher vegetation diversity was associated with lower Cd enrichment and mobility in soils. Except for BD, both total Cd and available Cd were highly negatively correlated with most soil physicochemical properties, including TPO, CP, AN, NH_4_^+^–N, NO_3_^−^–N. BD showed a significant positive correlation with both total Cd and available Cd, indicating that soil compaction enhanced Cd accumulation and increased its bioavailability.

**Figure 7:**
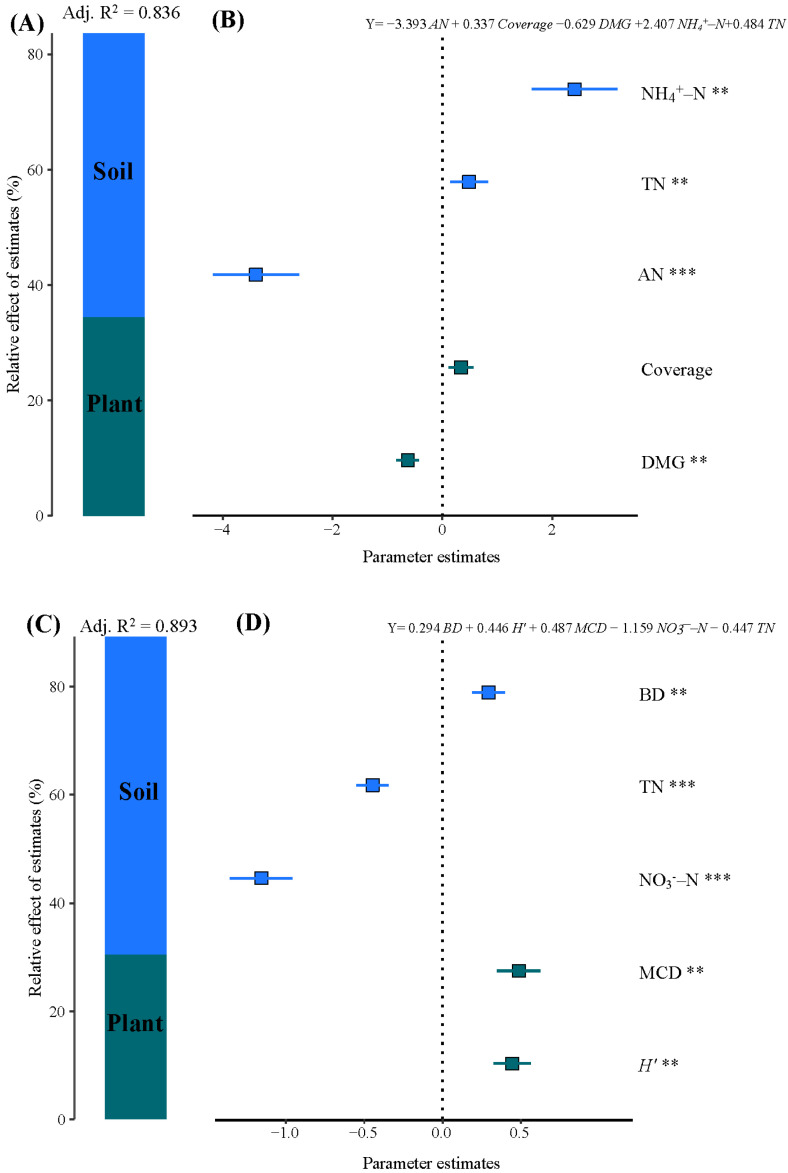
Relative effects of multiple predictors on total Cd and available Cd. Squares represent the averaged standardized regression coefficients, and horizontal lines indicate the corresponding 95% confidence intervals. The averaged parameter estimates (standardized regression coefficients) of the model predictors are shown with their associated 95% confidence intervals (**B**,**D**), along with the relative importance of each predictor, expressed as the percentage of explained variance (**A**,**C**) for total Cd and available Cd. The relative effect of the predictors is calculated as the ratio between the parameter estimate of the predictor and the sum of all parameter estimates, and it is expressed as a percentage. Soil properties include BD, total N (TN), ammonium nitrogen (NH_4_^+^–N), nitrate nitrogen (NO_3_^−^–N), and available N (AN); plant properties include vegetation coverage (Coverage), mean crown diameter (MCD), Margalef species richness index (DMG), and Shannon–Wiener index (*H*′). ** *p* < 0.01; *** *p* < 0.001.

Multiple linear regression (MLR) and hierarchical partitioning were used to explore potential predictors of total and available Cd concentrations in soil (Figure 7). For total Cd, AN, vegetation coverage, DMG, NH_4_^+^–N, and TN repeatedly appeared in the best-supported MLR models (ΔAIC < 2), indicating their relatively higher importance among the considered variables. The regression equation is expressed as follows:

(7) $$Total\;Cd=-3.393AN+0.337Coverage-0.629DMG+2.407NH_{4}^{+}-N+0.484TN$$

Among these variables, AN and DMG exhibited the strongest associations, showing opposite directions: AN negatively associated with soil total Cd, while NH_4_^+^–N and TN positively associated with soil total Cd. The hierarchical partitioning model yielded an adjusted R^2^ of 0.836, suggesting that the selected predictors jointly explained 83.6% of the total variance in total Cd (Figure 7A). Soil variables (AN, NH_4_^+^–N, and TN) contributed 49.21% of the explained variance, while plant community properties (DMG and coverage) accounted for 34.35%. These results indicate that soil N fractions explained a relatively large proportion of the variation in total Cd, while plant diversity exerted a moderate regulatory influence.

For available Cd, the best model incorporated BD, total N, nitrate nitrogen (NO_3_^−^–N), mean crown diameter (MCD), and Shannon–Wiener index (*H*′) (*p* < 0.01). The regression equation is as follows:

(8) $$Available\;Cd=0.294BD+0.446H^{\prime }+0.487MCD-1.159NO_{3}^{-}-N-0.447TN$$

In this model, BD, *H*′, and MCD showed positive effects on Cd bioavailability, while NO_3_^−^–N and TN exhibited negative effects (Figure 7D). The hierarchical partitioning analysis explained 89.3% of the total variation in available Cd (Adj. R^2^ = 0.893; Figure 7C). Soil properties contributed approximately 58.82% of the explained variance, suggesting that soil physicochemical factors played a major role in explaining Cd availability, whereas plant structural and diversity attributes contributed 30.44% of the variation. Together, these results suggest that soil N fractions and plant community composition jointly regulate both Cd accumulation and bioavailability in the restoration system.

## 4. Discussion

### 4.1. The Transport and Absorption of Cd by the Dominant Species

The dominant species varied among treatments, with *B. pilosa* and *Imperata cylindrica* in NR, *C. zizanioides* and *Medicago sativa* in PMC, and *M. sativa* and *E. curvula* in PME. The dominance of *B. pilosa* and *I. cylindrica* in NR suggests that under nutrient-poor conditions without human intervention, the plant community is dominated by drought- and stress-tolerant weeds. By contrast, both PMC and PME introduced a legume–grass mixture that may increase nutrient acquisition and biomass production, thereby potentially strengthening plant-mediated Cd capture and stabilization. As a leguminous plant, *Medicago sativa* can improve soil nitrogen availability through symbiotic N fixation with rhizobia, converting atmospheric N_2_ into ammonium [48], and potentially facilitating neighboring species by improving soil fertility and rhizosphere activity. Moreover, the rhizodeposition of amino acids and organic acids by *M. sativa* has been suggested to provide nutrients to neighboring and may contribute to the alleviation of Cd toxicity through root–root interactions, according to previous studies [49]. *Medicago sativis* [50], a taproot species that interacts, respectively, with the fibrous-rooted *C. zizanioides* [51] and *E. curvula* [52] may improve resource utilization and increase plant biomass through niche complementarity, and ultimately improve Cd pollution remediation [53]. This process may enhance its own biomass accumulation and resistance to heavy metal stress through the synthesis of chelating peptides and activation of antioxidant systems, as reported in previous studies [54]. For instance, Xiong et al. [48] found that the intercropping of *M. sativa* with *Ricinus communis* significantly improved the latter’s Cd and Zn extraction efficiency due to enhanced soil fertility, consistent with the facilitative role of *M. sativa* observed here. In this study, Cd concentrations were generally higher in roots than in shoots for all species, suggesting that root retention was a major pathway in the restored karst soils. Among the dominant species, *C. zizanioides* in PMC exhibited the highest Cd accumulation, with root and shoot Cd concentrations of 0.167 ± 0.020 mg/kg and 0.103 ± 0.011 mg/kg, respectively, and BCF and TF values of 2.10 ± 0.23 and 0.62 ± 0.05, respectively. This result suggests strong Cd enrichment in roots, consistent with Phusantisampan et al. [55], who emphasized that vetiver is an effective phytostabilizer capable of immobilizing Cd in roots. The large and fibrous root system of *C. zizanioides* provides vast surface area for metal adsorption and sequestration, which is consistent with its recognized phytostabilization potential [56]. In addition to physical adsorption, dense fibrous roots may enhance rhizosphere water retention and dampen short-term hydraulic fluctuations, thereby potentially limiting Cd leaching during rainfall pulses. This process is particularly relevant in karst regions, which are highly sensitive to climate extremes such as intensified diurnal temperature variability and altered wind regimes [8]. Recent evidence further suggests that Cd exposure may reshape the vetiver rhizosphere microbiome and stress-responsive transcription, supporting a rhizosphere-assisted stabilization mechanism rather than simple passive adsorption alone [57]. Therefore, the dense fibrous root system and enhanced rhizosphere recruitment of *C. zizanioides* facilitated stronger Cd stabilization and a more pronounced reduction in available Cd, which is likely associated with an advantage over PME. The BCF value of *C. zizanioides* in this study was higher than that reported by Ng and Abas [58], which may be explained by differences in soil pollution levels or co-cultivation with *M. sativa*, which may enhance Cd uptake via rhizosphere facilitation.

In conclusion, revegetation may promote community complexity and functional root traits that are important for Cd stabilization in the thin carbonate-rich soils typical of karst landscapes, where metal mobility is largely governed by rhizosphere processes [59] and soil structure rather than bulk chemistry alone [60]. The legume *M. sativis* can supply nutrients and rhizosphere carbon that may stimulate microbial activity and root growth, while *C. zizanioides* provides a dense root matrix that is favorable for Cd adsorption and physical immobilization.

### 4.2. Response of Community Functional Diversity to Soil Chemical Properties and Cd Concentration

Vegetation restoration markedly altered the community structure and diversity, which in turn was associated with improvements in soil physicochemical properties and reduced Cd contamination [61,62]. Compared with the NR, the composite restoration treatments (PMC and PME) supported higher plant abundance, richness, and evenness, indicating the establishment of a more complex and stable shrub–grass community structure. In particular, PMC exhibited the greatest species richness and *H*′, accompanied by the lowest ecological dominance, suggesting strong interspecific complementarity among shrub, leguminous, and grass species.

The results of the Pearson correlation heatmap showed that species richness, *H*′, and vegetation coverage were positively correlated with SOM, TN, and AP, whereas all diversity parameters were negatively correlated with BD, total cadmium, and available Cd. These relationships suggest that higher diversity may enhances soil nutrient and structure (lower BD), thereby potentially reducing Cd availability—an effect that is especially important in karst ecosystems characterized by thin soils, severe nutrient deficiency, and high vulnerability to degradation [63]. The negative correlations between all diversity parameters and BD (*p* < 0.05) indicate that the underground intricate root network of shrubs and herbs interpenetrate the soil of vegetation community structure, increasing soil pores and providing preferential flow paths, thereby reducing compaction and potentially improving water content [64]. This mechanism is consistent with the findings of Su et al. [65], who demonstrated that soil water retention characteristics and structural heterogeneity strongly regulate water and solute movement. The roots of shrubs are well developed [66], whereas those of herbaceous plants are dense [67], and both can continuously release various chemical substances (such as sugars and organic acids) throughout the year, significantly influencing soil aggregate structure and microbial activity [42]. Root exudates, litter inputs, and microbial activation are widely reported to promote aggregate formation and organic matter accumulation, which may contribute to reducing BD and improving porosity and aeration [68]. Root exudates can regulate the composition and functional performance of the soil microbial community, promoting the production of specific functional molecules (such as glomalin-related proteins and extracellular polymers), thereby enhancing the binding among soil particles [69]. Improvements in soil physical structure further facilitate nutrient retention and cycling [70]. Moreover, plant diversity increasing plant diversity was associated with enhanced biomass accumulation and distribution, as well as litter and root inputs and decomposition [71], thereby influencing soil nutrient status and fertility [72]. In the PMC and PME treatments, leguminous species enhanced NH_4_^+^–N and NO_3_^−^–N via N fixation, while grasses increased soil carbon inputs and were associated with Cd immobilization, which may have enhanced extracellular enzyme activity and nitrogen mineralization rate, thereby helping to explaining the simultaneous increase in NH_4_^+^–N and NO_3_^−^–N concentrations [73]. According to Wang et al. [74], vegetation restoration improves soil physicochemical properties and biotic functions; their study of spontaneous restoration of Pb–Zn mining wastelands showed that revegetation increased SOM and TN and decreased heavy metals, indicating that plant establishment and microbial interactions are associated with enhanced nutrient cycling and reduced metal mobility. Moreover, both total and available Cd concentrations decreased by nearly half under PMC compared with NR, indicating an apparent improvement in soil environmental quality following vegetation restoration and community diversification. However, this pronounced and rapid decline cannot be attributed solely to plant uptake and rhizosphere-mediated stabilization. In karst landscapes, strong spatial heterogeneity in parent material and soil geochemical background may generate substantial variability in baseline Cd concentrations, which could partially contribute to the observed treatment differences [75]. In addition, enhanced vegetation cover under composite restoration may reduce surface Cd concentrations through erosion control and lateral redistribution, particularly during intense rainfall events characteristic of subtropical karst regions [3]. The accumulation of soil organic matter following revegetation may also induce a dilution effect, whereby increases in soil mass and carbon inputs lower Cd concentrations on a mass basis without proportional changes in total Cd stocks [61]. Taken together, the pronounced decline in Cd under PMC likely reflects the combined effects of biological stabilization, altered soil physical structure, and landscape scale redistribution processes, rather than a single dominant mechanism.

Cd concentrations are closely linked to rhizosphere processes and microbial regulation [61]. Wu et al. [3] reported that vegetation restoration improved soil structure by increasing soil aggregates and was associated with reduced heavy metal concentrations through plant uptake and microbial biosorption. As suggested by previous studies, in karst soils, vegetation restoration may modified rhizosphere chemistry, shifted Cd toward more stable residual and carbonate-bound fractions [76]. Previous studies have shown that diverse plant communities also increased microbial metabolism and enzyme activities (urease, phosphatase, catalase), thereby potentially enhancing Cd adsorption and deposition [74]. Similarly, Wu et al. [3] confirmed that root exudate and aggregate formation were jointly associated with reduced Ni, Pb, and Cd activity and improved soil stability. Total and available Cd decreased by 50% in PMC, highlighting the potential phytostabilization and ecological risk mitigation potential of diverse shrub–grass systems in karst environments. Our findings also align with other karst restoration studies showing that vegetation recovery may reduce soil bulk density and improve soil nutrient status, microbial functioning, and Cd immobilization more effectively than natural succession in rocky desertification landscapes [11,40,77]. However, microbial-mediated mechanisms remain underexplored. Insights from disturbance–recovery studies indicate that microbial communities may maintain functional resilience under coupled climatic and edaphic stress. For instance, Liu et al. [78] demonstrated that ecosystem recovery following environmental perturbation is strongly mediated by microbial functional stability, offering a useful parallel for understanding Cd immobilization in stressed karst ecosystems.

Beyond edaphic constraints, karst ecosystems are climatically vulnerable, and vegetation–soil feedbacks may modulate drought–flood alternation by improving infiltration and water holding capacity, thereby influencing nutrient cycling and, potentially, metal mobility [63]. At broader spatial scales, vegetation restoration may contributes to climate and hydrology feedbacks through altered evapotranspiration and terrestrial water cycling, as demonstrated by global greening studies [9,79], providing an important climatic context for interpreting local Cd stabilization.

Model-averaged regression and hierarchical partitioning identified the dominant drivers, indicating that soil properties were associated with a stronger control of total and available Cd concentrations than plant community attributes. Nutrient availability also appeared to modulated Cd accumulation. MLR identified AN, coverage, DMG, NH_4_^+^–N, and TN as significant predictors for total Cd (*p* < 0.01). The negative effect of AN on total Cd may be related to active N availability stimulates microbial processing and extracellular enzyme activity that can foster cadmium complexation with organic ligands and mineral surfaces, thereby potentially promoting Cd into less mobile soil pools [61,80]. The positive effect of NH_4_^+^–N on total Cd may be attributed to the nitrification process induced by NH_4_^+^–N, which releases H^+^ and decreases rhizosphere pH, thereby potentially causing Cd to desorb from carbonate or oxide binding sites [81]. For available Cd, the best MLR model included BD, TN, NO_3_^−^–N, MCD, and *H*′ (*p* < 0.01). This observation may be explained mechanistically: NO_3_^−^–N uptake by plants is commonly associated with rhizosphere alkalinization (relative to NH_4_^+^–N uptake), and a higher rhizosphere pH can reduce Cd solubility and favors adsorption to mineral and organic binding sites, thereby reducing the content of available Cd [81]. For example, Hu et al. [82] and Bai et al. [83] reported nitrate-facilitated Cd uptake or increased plant Cd accumulation for specific species, which is consistent with nitrate increasing root uptake fluxes, even while reducing extractable soil pools via plant removal. The role of nitrogen forms in Cd mobility has been widely discussed, and comparisons within coupled nutrient–metal systems provide additional mechanistic insight. Wang et al. [84] showed that coordinated regulation of nitrogen transformations can simultaneously suppress arsenic mobilization and N_2_O emissions, offering a valuable conceptual framework for interpreting coupled N–Cd dynamics in restored soils. Higher BD reduces macro porosity and gas diffusion, creating a low-oxygen environment that may promote microbially mediated reduction and dissolution of Fe/Mn oxides, thereby potentially releasing adsorbed Cd into the soil and increasing the content of available Cd [70,85]. Thus, the coupling of plant diversity, soil nutrient dynamics likely jointly governs Cd mobility and stabilization in restored karst soils, reinforcing the ecological benefits of multi-species revegetation.

From an application perspective, this provides clear practical meaning for karst restoration: PMC-type shrub–legume–vetiver assemblies may represent implementable nature-based solutions that can help stabilize fragile soils and mitigate Cd ecological risk by lowering phytoavailable Cd rather than relying solely on harvest-based removal.

## 5. Conclusions

This study shows that vegetation restoration was associated with higher plant species diversity, vegetation cover, and the productivity of newly established plant communities, as well as higher soil nutrient levels and lower concentrations of total and available Cd. These patterns suggest a close relationship between plant species diversity and ecological restoration in karst regions. Compared with natural recovery, both shrub–grass mixtures were associated with improved soil conditions and reduced Cd, with PMC (*Pistacia weinmannifolia* + *Medicago sativa* + *Chrysopogon zizanioides*) performing the best, reducing total Cd by 49.4% and available Cd by 59.5%. Soil AN, NH_4_^+^–N, TN, vegetation coverage, and Margalef species richness (DMG) were the main factors affecting soil total Cd content. Soil BD, TN, NO_3_^−^–N, MCD, and *H*′ were the main factors affecting soil available Cd content. Based on these results, PMC may represent a promising plant community configuration for the long-term phytostabilization of Cd-contaminated karst soils. Future studies should improve research quality by integrating advanced analytical approaches. Synchrotron-based spectroscopic techniques (X-ray absorption fine structure), high-throughput sequencing of rhizosphere microbial communities, and isotope tracing (^15^N) can be applied to identify Cd binding forms in soils, clarify microbial contributions to Cd stabilization, and quantify vegetation-mediated nitrogen transformation, respectively. In addition, long-term monitoring and field-scale validation should be incorporated to enhance early-warning capacity and ensure the sustainable application of phytostabilization in karst ecosystems.

## Figures and Tables

**Table 1:** Experimental treatments.

Treatment	Code	Sample
Natural recover	NR	S1, S5, S8
*Pistacia weinmanniifolia* J. Poiss. + *Medicago sativa* cv. WL525HQ + *Chrysopogon zizanioides* (L.) Roberty	PMC	S2, S4, S7
*Pistacia weinmanniifolia* J. Poiss. + *Medicago sativa* cv. WL525HQ. + *Eragrostis curvula* (Schrad.) Nees	PME	S3, S6, S9

**Table 2:** Species composition (n m^−2^) of three treatments.

Plant Species and Community Characteristics	NR			PMC			PME		
	S1	S5	S8	S2	S4	S7	S3	S6	S9
*Imperata cylindrica* (L.) Raeusch.	17	19	6	–	8	4	–	5	–
*Miscanthus sinensis* Anderss.	5	4	–	10	–	–	–	–	6
*Bidens pilosa* L.	3	–	12	2	–	–	2	–	–
*Medicago sativa cv.* WL525HQ	–	–	–	36	29	43	44	40	47
*Eragrostis curvula* (Schrad.) Nees	–	–	–	–	–	–	24	18	20
*Chrysopogon zizanioides* (L.) Roberty	–	–	–	19	16	21	–	–	–
*Kummerowia striata* (Thunb.) Schindl.	–	–	–	–	–	3	–	–	–
*Erigeron canadensis* L.	–	–	6	6	–	–	13	–	11
*Praxelis clematidea*	–	–	–	–	–	5	–	–	–
*Pistacia weinmanniifolia* J. Poiss.	–	–	–	2	2	2	2	3	3
*Leucaena leucocephala* (Lam.) de Wit	–	–	–	–	–	1	–	–	1
*Photinia serratifolia* (Desf.) Kalkman	–	–	–	–	1	–	2	–	–
*Salix myrtillacea* Andersson	–	–	–	1	–	–	–	–	–
*Breynia fruticosa* L.	–	–	–	–	–	–	–	–	–
*Oxalis corniculata* L.	–	–	–	–	5	–	–	–	–
*Heteropogon contortus* (L.) Beauv. ex Roem. & Schulz.	–	–	–	–	–	–	6	–	–
*Inula cappa* (Buch.-Ham. ex D.Don) DC.	–	–	–	–	–	–	–	12	–
Total numbers of individuals (n, n·m^−2^)	25	23	24	76	61	80	93	78	88

Note: “–” indicates absence of the species in the corresponding plot.

**Table 3:** Physicochemical properties and Cd contents of surface soil from different treatments.

Factors	NR	PMC	PME
BD (g cm^−3^)	1.64 ± 0.12 ^a^	1.27 ± 0.06 ^b^	1.35 ± 0.14 ^b^
CP (%)	16.6 ± 1.2 ^c^	23.5 ± 0.4 ^a^	21.1 ± 1.1 ^b^
NCP (%)	6.6 ± 0.9 ^a^	7.0 ± 0.7 ^a^	7.5 ± 0.7 ^a^
TPO (%)	23.1 ± 0.5 ^c^	30.5 ± 0.3 ^a^	28.6 ± 1.4 ^b^
SOM (g·kg^−1^)	10.21 ± 0.09 ^b^	13.59 ± 1.08 ^a^	10.05 ± 0.27 ^b^
TN (g·kg^−1^)	1.23 ± 0.04 ^b^	1.39 ± 0.02 ^a^	1.34 ± 0.07 ^a^
TP (g·kg^−1^)	0.444 ± 0.022 ^c^	0.561 ± 0.049 ^b^	0.735 ± 0.058 ^a^
AN (mg kg^−1^)	53.45 ± 6.63 ^c^	114.53 ± 12.13 ^a^	81.00 ± 5.08 ^b^
AP (mg kg^−1^)	22.55 ± 1.10 ^c^	23.75 ± 0.72 ^b^	25.56 ± 1.08 ^a^
NH_4_^+^–N (mg kg^−1^)	7.88 ± 0.48 ^c^	24.41 ± 1.95 ^a^	15.95 ± 1.82 ^b^
NO_3_^−^–N (mg kg^−1^)	27.66 ± 2.69 ^c^	47.88 ± 2.10 ^a^	41.77 ± 1.12 ^b^
Total Cd (mg kg^−1^)	0.156 ± 0.024 ^a^	0.079 ± 0.009 ^c^	0.101 ± 0.014 ^b^
Available Cd (mg kg^−1^)	0.042 ± 0.007 ^a^	0.017 ± 0.003 ^c^	0.027 ± 0.003 ^b^

Note: BD: Bulk density; CP: capillary porosity; NCP: non capillary porosity; TPO: total porosity; SOM: soil organic matter; TN: total nitrogen; TP: total phosphorus; AN: alkali-hydrolyzable nitrogen; AP: available phosphorus; NH_4_^+^–N: ammonium nitrogen; NO_3_^−^–N: nitrate nitrogen; Total Cd: total cadmium in soil; Available Cd: available cadmium. The values are means ± SD of three replicates for each treatment. Different letters indicate significant differences among species at same plant parts according to Duncan at *p* < 0.05.

## Data Availability

The data presented in this study are available upon request from the corresponding authors.

## Appendix A

**Table A1:** Importance values (IV, %) of plant species under different treatments (mean ± SD).

Treatment	Species	IV (%)
BL	*Imperata cylindrica*	65.89 ± 34.46 ^a^
BL	*Bidens* *p* *ilosa*	30.24 ± 13.09 ^b^
BL	*Erigeron canadensis*	19.53 ^bc^
BL	*Miscanthus sinensis*	11.15 ± 0.36 ^c^
PMC	*Chrysopogon zizanioides*	58.11 ± 2.76 ^a^
PMC	*Medicago sativa*	30.69 ± 2.03 ^b^
PMC	*Miscanthus sinensis*	6.88 ^c^
PMC	*Imperata cylindrica*	4.99 ± 2.91 ^c^
PMC	*Oxalis corniculata*	5.01 ^c^
PMC	*Erigeron canadensis*	4.28 ^c^
PMC	*Praxelis clematidea*	4.13 ^c^
PMC	*Kummerowia striata*	2.00 ^c^
PMC	*Bidens pilosa*	1.39 ^c^
PME	*Medicago sativa*	59.66 ± 5.82 ^a^
PME	*Eragrostis curvula*	22.21 ± 2.39 ^b^
PME	*Heteropogon contortus*	17.44 ^c^
PME	*Inula cappa*	11.71 ^c^
PME	*Erigeron canadensis*	7.84 ± 0.47 ^cd^
PME	*Imperata cylindrica*	4.51 ^d^
PME	*Miscanthus sinensis*	3.75 ^d^
PME	*Bidens pilosa*	1.25 ^d^

The values are means ± SD of three replicates for each treatment. Different letters indicate significant differences among species at same plant parts according to Duncan at *p* < 0.05.

## References

[1] Rahim H.U., Akbar W.A., Alatalo J.M. (2022). A Comprehensive Literature Review on Cadmium (Cd) Status in the Soil Environment and Its Immobilization by Biochar-Based Materials. Agronomy.

[2] Su C., Wang J., Chen Z., Meng J., Yin G., Zhou Y., Wang T. (2023). Sources and Health Risks of Heavy Metals in Soils and Vegetables from Intensive Human Intervention Areas in South China. Sci. Total Environ..

[3] Wu Y., Tian X., Wang R., Zhang M., Wang S. (2023). Effects of Vegetation Restoration on Distribution Characteristics of Heavy Metals in Soil in Karst Plateau Area of Guizhou. PeerJ.

[4] Firmani G., Chiavarini M., Dolcini J., Quarta S., D’Errico M.M., Barbadoro P. (2024). The Association between Cadmium Exposure and Prostate Cancer: An Updated Systematic Review and Meta-Analysis. Int. J. Environ. Res. Public Health.

[5] Zhou Z., Shi Z., Yu L., Fan H., Wan F. (2025). Soil Quality and Heavy Metal Source Analyses for Characteristic Agricultural Products in Luzuo Town, China. Agriculture.

[6] Lin G., Zhang C., Yang Z., Li Y., Liu C., Ma L.Q. (2024). High Geological Background Concentrations of As and Cd in Karstic Soils May Not Contribute to Greater Risks to Human Health via Rice Consumption. J. Hazard. Mater..

[7] Zhang J., Chen H., Fu Z., Wang K. (2021). Effects of Vegetation Restoration on Soil Properties along an Elevation Gradient in the Karst Region of Southwest China. Agric. Ecosyst. Environ..

[8] Sun R., Wu X. (2026). Extremes of Diurnal Temperature Range in Karst Regions: Definition, Trends and Interaction with Wind Speed. Atmos. Res..

[9] Chai Y., Miao C., Slater L., Ciais P., Berghuijs W.R., Chen T., Huntingford C. (2025). Underestimating Global Land Greening: Future Vegetation Changes and Their Impacts on Terrestrial Water Loss. One Earth.

[10] Wen Y., Wang Y., Ji W., Wei N., Liao Q., Huang D., Meng X., Song Y. (2023). Influencing Factors of Elevated Levels of Potentially Toxic Elements in Agricultural Soils from Typical Karst Regions of China. Agronomy.

[11] Tan S., Zhang Z., Zhou L., Li Y., Lu S., Tang C., Yu L. (2025). Natural Vegetation Restoration Promotes Soil Quality Improvement in Rocky Desertification Areas of Southwestern China. Plant Soil.

[12] Zhu G., Zhao J., Chen Q., Guo Q., Cheng D., Bijaya G.C.D., Li W. (2022). The Comparative Potential of Four Compositae Plants for Phytoremediation of Karst Lead/Zinc Mine Tailings Contaminated Soil. BioResources.

[13] Wang W., Xue J., Zhang L., He M., Cai R., You J. (2025). Phytoremediation as a Sustainable Tool to Rehabilitate Land Contaminated by High-Density Sludge Sediment: From Waste to Green. Plant Soil.

[14] Han Y., Wang H., Zhang G., Zhang S., Liu X., Liu L. (2022). Distribution, Ecological Risk Assessment and Source Identification of Pollutants in Soils of Different Land-Use Types in Degraded Wetlands. PeerJ.

[15] Sun C., Zhu S., Zhao B., Li W., Gao X., Wang X. (2020). Effect of Land Use Conversion on Surface Soil Heavy Metal Contamination in a Typical Karst Plateau Lakeshore Wetland of Southwest China. Int. J. Environ. Res. Public Health.

[16] Zhang B., Hu G., Xu C., Hu C., Zhong C., Chen S., Zhang Z. (2024). Effects of Natural Vegetation Restoration on Soil Physicochemical Properties in Tropical Karst Areas, Southwestern China. Forests.

[17] Jia P., Liang J., Yang S., Zhang S., Liu J., Liang Z., Li F., Zeng Q., Fang Z., Liao B. (2020). Plant Diversity Enhances the Reclamation of Degraded Lands by Stimulating Plant–Soil Feedbacks. J. Appl. Ecol..

[18] Sun C., Li F., He X., Qian Z., Qin Y. (2024). Positive Effects of Plant Species Diversity on Organic Carbon Accumulation in Soil Aggregates Driven by Mineral Protection in a Subtropical Forest in Southwest China. J. Soils Sediments.

[19] Zhu G., Shangguan Z., Deng L. (2021). Dynamics of Water-Stable Aggregates Associated Organic Carbon Assessed from Delta C-13 Changes Following Temperate Natural Forest Development in China. Soil Tillage Res..

[20] Wang J., Lu X., Zhang J., Ouyang Y., Wei G., Xiong Y. (2020). Rice Intercropping with Alligator Flag (*Thalia dealbata*): A Novel Model to Produce Safe Cereal Grains While Remediating Cadmium Contaminated Paddy Soil. J. Hazard. Mater..

[21] Wieczorek J., Baran A., Bubak A. (2023). Mobility, Bioaccumulation in Plants, and Risk Assessment of Metals in Soils. Sci. Total Environ..

[22] Wu Q., Zheng W., Rao C., Wang E., Yan W. (2022). Soil Quality Assessment and Management in Karst Rocky Desertification Ecosystem of Southwest China. Forests.

[23] Zheng W., Wu Q., Guo X., Zhou P., Wu J., Yan W. (2024). Rocky Desertification Succession Alters Soil Microbial Communities and Survival Strategies in the Karst Context. Sci. Total Environ..

[24] Hu Z., Wu Z., Luo W., Liu S., Tu C. (2024). Spatial Distribution, Risk Assessment, and Source Apportionment of Soil Heavy Metals in a Karst County Based on Grid Survey. Sci. Total Environ..

[25] Zhu G., Xiao H., Guo Q., Song B., Zheng G., Zhang Z., Zhao J., Okoli C.P. (2018). Heavy Metal Contents and Enrichment Characteristics of Dominant Plants in Wasteland of the Downstream of a Lead-Zinc Mining Area in Guangxi, Southwest China. Ecotoxicol. Environ. Saf..

[26] Sharma J.K., Kumar N., Singh N.P., Santal A.R. (2023). Phytoremediation Technologies and Their Mechanism for Removal of Heavy Metal from Contaminated Soil: An Approach for a Sustainable Environment. Front. Plant Sci..

[27] Xu T., Wu X., Tian Y., Li Y., Zhang W., Zhang C. (2021). Soil Property Plays a Vital Role in Vegetation Drought Recovery in Karst Region of Southwest China. J. Geophys. Res. Biogeosci..

[28] Zou X., Yao K., Zeng Z., Zeng F., Lu L., Zhang H. (2024). Effect of Different Vegetation Restoration Patterns on Community Structure and Co-Occurrence Networks of Soil Fungi in the Karst Region. Front. Plant Sci..

[29] Huang Y., Ma R., Shi H., Li J., Tu S. (2023). Centennial Lake Environmental Evolution Reflected by Diatoms in Yilong Lake, Yunnan Province, China. Appl. Sci..

[30] Xiao C., You R., Zhu N., Mi X., Gao L., Zhou X., Zhou G. (2023). Variation of Soil Physicochemical Properties of Different Vegetation Restoration Types on Subtropical Karst Area in Southern China. PLoS ONE.

[31] Choi M.K., Azeez B.S., Lee S.W., Li W.Y., Choi S., Choi I.-Y., Choi K.-Y., Na J.-K. (2024). Transcriptome Analysis of a Tropical Medicinal Plant, *Pistacia weinmannifolia*. J. Plant Biotechnol..

[32] Song X., Yuan Z.-Q., Fang C., Hu Z.-H., Li F.-M., Sardans J., Penuelas J. (2024). The Formation of Humic Acid and Micro-Aggregates Facilitated Long-Time Soil Organic Carbon Sequestration after *Medicago Sativa* L. Introduction on Abandoned Farmlands. Geoderma.

[33] Zhang Y., Wang L. (2025). Advances in Basic Biology of Alfalfa (*Medicago sativa* L.): A Comprehensive Overview. Hortic. Res..

[34] Chen X.W., Wong J.T.F., Wang J.-J., Wong M.H. (2021). Vetiver Grass-Microbe Interactions for Soil Remediation. Crit. Rev. Environ. Sci. Technol..

[35] Motsomane N., Magadlela A. (2025). Eragrostis Curvula Cultivars Improve Soil Bacterial Diversity, Extracellular Enzyme Activities, and Nutrition in Grassland Ecosystem Soils. Sci. Rep..

[36] Ren L., Huo J., Xiang X., Pan Y., Li Y., Wang Y., Meng D., Yu C., Chen Y., Xu Z. (2023). Environmental Conditions Are the Dominant Factor Influencing Stability of Terrestrial Ecosystems on the Tibetan Plateau. Commun. Earth Environ..

[37] Chen X., Hein P.P., Shi M., Yang F., Yang J., Fu Y., Yang X. (2024). Diversity and Traditional Knowledge Concerning Fodder Plants Are Invaluable Assets for Enhancing the Sustainable Management of Crop-Livestock System of Zhaotong City in the Mountainous Southwest China. Plant Divers..

[38] Xu G., Mo Q., Li Z., Qin W., Dong R., Zhao X., Chen C., Gunina A., Shurpali N., Thentu T.L. (2025). Integrated Physical–Chemical Mechanisms Drive Carbon Stabilization under Conservation Tillage in Karst Agroecosystems. CATENA.

[39] Chang H., Zhang C., Zang S., Zhu Y., Zhao W., Qu Y., Wang H. (2024). Study on the Influencing Mechanism of Biochar and Ethylenediaminetetraacetic Acid Combination on the Remediation of Cd Polluted Soil by *Sedum alfredii* Hance. Environ. Technol. Innov..

[40] Ou H.-B., Liu X.-S., Wei S.-X., Jiang Y., Gao F., Wang Z.-H., Fu W., Du H. (2024). The Effects of Different Vegetation Restoration Models on Soil Quality in Karst Areas of Southwest China. Forests.

[41] Kama R., Li S., Nabi F., Aidara M., Huang P., Li Z., Diatta S., Ma C., Li H. (2024). Hyperaccumulators’ Diversity Enhances Cd-Contaminated Soil Restoration and Reduces Rice Cd Uptake under an Intercropping System. ACS Omega.

[42] Zeng P., Guo Z., Xiao X., Peng C. (2019). Dynamic Response of Enzymatic Activity and Microbial Community Structure in Metal(Loid)-Contaminated Soil with Tree-Herb Intercropping. Geoderma.

[43] Guo N., Xie M., Fang Z., Jiao F., Han X. (2022). Divergent Responses of Plant Biomass and Diversity to Short-Term Nitrogen and Phosphorus Addition in Three Types of Steppe in Inner Mongolia, China. Ecol. Process..

[44] Cai Q.Y., Li B., Cai M.T., Liu Y.Z., Wu L., Ge G. (2023). Diversity and Distribution of Bryophytes in Ion-Type Rare Earth Mines in Southern Jiangxi Province, Southeast China, and the Implications for Vegetation Restoration. Plant Soil.

[45] Marabesi A.O., Lessl J.T., Coolong T.W. (2023). Cadmium Bioconcentration and Translocation Potential in Day Neutral and Photoperiod Sensitive Hemp Grown Hydroponically for the Medicinal Market. Water.

[46] Roebuck C.J., Klink M.J. (2025). Phytoremediation Potential of Hemp in Metal-Contaminated Soils: Soil Analysis, Metal Uptake, and Growth Dynamics. Processes.

[47] Wang C., Wang X., Zhang Y., Morrissey E., Liu Y., Sun L., Qu L., Sang C., Zhang H., Li G. (2023). Integrating Microbial Community Properties, Biomass and Necromass to Predict Cropland Soil Organic Carbon. ISME Commun..

[48] Xiong P., He C., Oh K., Chen X., Liang X., Liu X., Cheng X., Wu C., Shi Z. (2018). *Medicago sativa* L. Enhances the Phytoextraction of Cadmium and Zinc by *Ricinus communis* L. on Contaminated Land in Situ. Ecol. Eng..

[49] Ketaubon P., Ritthikasem N., Tanheng P., Prapagdee B. (2024). Enhancing Heavy Metal Phytoremediation in Landfill Soil by *Chrysopogon zizanioides* (L.) Roberty through the Application of Bacterial-Biochar Pellets. Environ. Technol. Innov..

[50] Li S., Wan L., Nie Z., Li X. (2020). Fractal and Topological Analyses and Antioxidant Defense Systems of Alfalfa (*Medicago sativa* L.) Root System under Drought and Rehydration Regimes. Agronomy.

[51] Holanda F.S.R., Santos L.D.V., Pedrotti A., De Araújo Filho R.N., Sartor L.R., Santos-Sobrinho V.R.A., De Jesus R.J.S., De Oliveira Silva P.A., Andrade K.M.A. (2022). Evaluation of the Root System of Vetiver Grass (*Chrysopogon zizanioides* L. Roberty) Using Different Sampling Methods. Environ. Syst. Res..

[52] Gomes P.I.A., Asaeda T. (2009). Spatial and Temporal Heterogeneity of *Eragrostis curvula* in the Downstream Flood Meadow of a Regulated River. Ann. Limnol. Int. J. Limnol..

[53] Williams L.J., Butler E.E., Cavender-Bares J., Stefanski A., Rice K.E., Messier C., Paquette A., Reich P.B. (2021). Enhanced Light Interception and Light Use Efficiency Explain Overyielding in Young Tree Communities. Ecol. Lett..

[54] Pacheco-Insausti M.C., Ponce I.T., Quiñones M.A., Pedranzani H.E., Pueyo J.J. (2023). Effects of Inoculation with Stress-Tolerant Rhizobia on the Response of Alfalfa (*Medicago sativa* L.) to Combined Salinity and Cadmium Stress. Plants.

[55] Phusantisampan T., Meeinkuirt W., Saengwilai P., Pichtel J., Chaiyarat R. (2016). Phytostabilization Potential of Two Ecotypes of *Vetiveria Zizanioides* in Cadmium-Contaminated Soils: Greenhouse and Field Experiments. Environ. Sci. Pollut. Res..

[56] Abaga N.O.Z., Dousset S., Munier-Lamy C. (2021). Phytoremediation Potential of Vetiver Grass (*Vetiveria zizanioides*) in Two Mixed Heavy Metal Contaminated Soils from the Zoundweogo and Boulkiemde Regions of Burkina Faso (West Africa). J. Geosci. Environ. Prot..

[57] Wu B., Li J., Peng D., Wang Z., Xu H. (2022). Cadmium Exposure Alters Rhizospheric Microbial Community and Transcriptional Expression of Vetiver Grass. Front. Plant Sci..

[58] Ng C.C., Abas M.R. (2017). Tolerance Threshold and Phyto-Assessment of Cadmium and Lead in Vetiver Grass, *Vetiveria zizanioides* (Linn.) Nash. Chiang Mai J. Sci..

[59] Cheng L., Li Z., Zhou L., Xie J., Zhou Q., Ding M., Wang P., Zhang H., Nie M., Huang G. (2025). Rhizosphere Microbiota Modulate Cadmium Mobility Dynamics and Phytotoxicity in Rice under Differential Cd Stress. Plant Soil.

[60] Lan J., Jiang Y., Huang M. (2025). Vegetation Restoration Enhances Soil Organic Carbon Accumulation in Southwest China’s Karst Region: The Role of Aggregation, Calcium, and Microbes. CATENA.

[61] Chen J., Hao W., Shi Y., Chen L., Li H., Zhao Z., Mo M., Li T. (2025). Phytomanagement with Forage Grasses for Sustainable Remediation of Contaminated Tailings Soil: Enhancing Soil Functionality and Addressing Forage Safety Risks. Environ. Technol. Innov..

[62] Yuan X., Guo Z., Wang S., Zhao L., Yuan M., Gao Y., Huang L., Liu C., Duan C. (2023). Drivers and Mechanisms of Spontaneous Plant Community Succession in Abandoned Pb Zn Mining Areas in Yunnan, China. Sci. Total Environ..

[63] He B., Li Q., Zou S., Bai X., Li W., Chen Y. (2024). Dynamic Changes of Soil Microbial Communities during the Afforestation of Pinus Armandii in a Karst Region of Southwest China. Microb. Ecol..

[64] Peng X., Dai Q., Ding G., Shi D., Li C. (2019). The Role of Soil Water Retention Functions of Near-Surface Fissures with Different Vegetation Types in a Rocky Desertification Area. Plant Soil.

[65] Su Y., Cui Y.-J., Dupla J.-C., Canou J. (2022). Soil-Water Retention Behaviour of Fine/Coarse Soil Mixture with Varying Coarse Grain Contents and Fine Soil Dry Densities. Can. Geotech. J..

[66] Dong L., Liu Y., Wu J., Liao Y., Li J., Yu J., Wang S., Yu Z., Shangguan Z., Deng L. (2023). The Distribution of Soil C and N along the Slope Is Regulated by Vegetation Type on the Loess Plateau. CATENA.

[67] Zhang S., Xiao Z., Huo J., Zhang H. (2021). Key Factors Influencing on Vegetation Restoration in the Gullies of the Mollisols. J. Environ. Manag..

[68] Lu Z.-X., Wang P., Ou H.-B., Wei S.-X., Wu L.-C., Jiang Y., Wang R.-J., Liu X.-S., Wang Z.-H., Chen L.-J. (2022). Effects of Different Vegetation Restoration on Soil Nutrients, Enzyme Activities, and Microbial Communities in Degraded Karst Landscapes in Southwest China. For. Ecol. Manag..

[69] Angst Š., Angst G., Mueller K.E., Lange M., Eisenhauer N. (2025). Un(Der)Explored Links between Plant Diversity and Particulate and Mineral-Associated Organic Matter in Soil. Nat. Commun..

[70] Xu L., Xing X., Bai J., Li D. (2022). Soil Aggregate Structure, Stability, and Stoichiometric Characteristics in a Smelter-Impacted Soil under Phytoremediation. Front. Environ. Sci..

[71] Zhang Y., Xu X., Li Z., Liu M., Xu C., Zhang R., Luo W. (2019). Effects of Vegetation Restoration on Soil Quality in Degraded Karst Landscapes of Southwest China. Sci. Total Environ..

[72] Yan Z., Zhou J., Liu C., Jia R., Mganga K.Z., Yang L., Yang Y., Peixoto L., Zang H., Zeng Z. (2023). Legume-Based Crop Diversification Reinforces Soil Health and Carbon Storage Driven by Microbial Biomass and Aggregates. Soil Tillage Res..

[73] Liu L., Zhu Q., Wen D., Yang L., Ni K., Xu X., Cao J., Meng L., Yang J., Zhou J. (2024). Stimulation of Organic N Mineralization by N–acquiring Enzyme Activity Alleviates Soil Microbial N Limitation Following Afforestation in Subtropical Karst Areas. Plant Soil.

[74] Wang S., Li T., Yuan X., Yu J., Luan Z., Guo Z., Yu Y., Liu C., Duan C. (2025). Biotic and Abiotic Drivers of Soil Carbon, Nitrogen and Phosphorus and Metal Dynamic Changes during Spontaneous Restoration of Pb–Zn Mining Wastelands. J. Hazard. Mater..

[75] Wang Y., Jing J., Li Y., Zhang Y., Liu Y. (2025). Bioavailability and Speciation of Potentially Toxic Trace Metals in Limestone-Derived Soils in a Karst Region, Southwestern China. Water. Air. Soil Pollut..

[76] Li J., Huang C., Huang Z., Wang X., Luo J., Feng S., Yang Z. (2024). Exploring the Geochemical Characteristics, Sources, Influencing Factors, and Potential Remediation Strategies of Cd in a Typical Karst Region. Environ. Earth Sci..

[77] Zheng W., Guo X., Zhou P., Tang L., Lai J., Dai Y., Yan W., Wu J. (2024). Vegetation Restoration Enhancing Soil Carbon Sequestration in Karst Rocky Desertification Ecosystems: A Meta-Analysis. J. Environ. Manag..

[78] Liu Y., Qiu H., Wang N., Yang D., Zhao K., Yang G., Huangfu W., Luo W. (2025). Thermokarst Disturbance Responses to Climate Change across the Circumpolar Permafrost Regions from 1990 to 2023. Geosci. Front..

[79] Yu L., Liu Y., Liu T., Yan F. (2020). Impact of Recent Vegetation Greening on Temperature and Precipitation over China. Agric. For. Meteorol..

[80] Zhang Y., Zhen Q., Ma W., Jia J., Li P., Zhang X. (2023). Dynamic Responses of Soil Aggregate-Associated Organic Carbon and Nitrogen to Different Vegetation Restoration Patterns in an Agro-Pastoral Ecotone in Northern China. Ecol. Eng..

[81] Huo W., Zou R., Wang L., Guo W., Zhang D., Fan H. (2018). Effect of Different Forms of N Fertilizers on the Hyperaccumulator *Solanum Nigrum* L. and Maize in Intercropping Mode under Cd Stress. RSC Adv..

[82] Hu P., Yin Y.-G., Ishikawa S., Suzui N., Kawachi N., Fujimaki S., Igura M., Yuan C., Huang J., Li Z. (2013). Nitrate Facilitates Cadmium Uptake, Transport and Accumulation in the Hyperaccumulator Sedum Plumbizincicola. Environ. Sci. Pollut. Res..

[83] Bai Z., Li D., Zhu L., Tang X., Wang Y., Mao R., Wu J. (2021). Nitrate Increases Cadmium Accumulation in Sweet Sorghum for Improving Phytoextraction Efficiency Rather than Ammonium. Front. Plant Sci..

[84] Wang F., Zhang J., Hu J., Wang H., Zeng Y., Wang Y., Huang P., Deng H., Dahlgren R.A., Gao H. (2024). Simultaneous Suppression of as Mobilization and N_2_O Emission from NH_4_^+^/as-Rich Paddy Soils by Combined Nitrate and Birnessite Amendment. J. Hazard. Mater..

[85] Liu S., Lin Z., Duan X., Deng Y. (2024). Effects of Soil Microorganisms on Aggregate Stability during Vegetation Recovery in Degraded Granitic Red Soil Areas. Appl. Soil Ecol..

